# Supplementary material for: Shape-Memory Composites Based on Ionic Elastomers
Source: Polymers (Basel). 2022 Mar 18;14(6):1230. doi: 10.3390/polym14061230 (PMC8953204; doi:10.3390/polym14061230)
Supplement: Supplementary file 1 [file polymers-14-01230-s001.zip › manuscript-supplementary.pdf]

Supporting information

Shape-Memory Composites Based on Ionic Elastomers

Antonio González-Jiménez, Pilar Bernal-Ortega, Fernando M. Salamanca and Juan L. Valentin

TGA

Thermogravimetric analysis (TGA) was carried out on a TA discovery (TA Instruments, USA), by equilibrating at 100 °C for 20 min and with a heating ramp of 10 °C/min under a N_2 atmosphere up to 650 °C and O_2 atmosphere up to 1000 °C.

To verify the correct incorporation of the different fillers to the matrix, a thermogram of the study samples is represented in Figure S1. Two differentiated processes can be seen in these thermograms: (i) weight loss up to 400 ° C corresponding to the degradation of the elastomeric matrix; And (ii) the decomposition temperature of the different carbonaceous fillers, corresponding to the degradation of the main carbon structure.

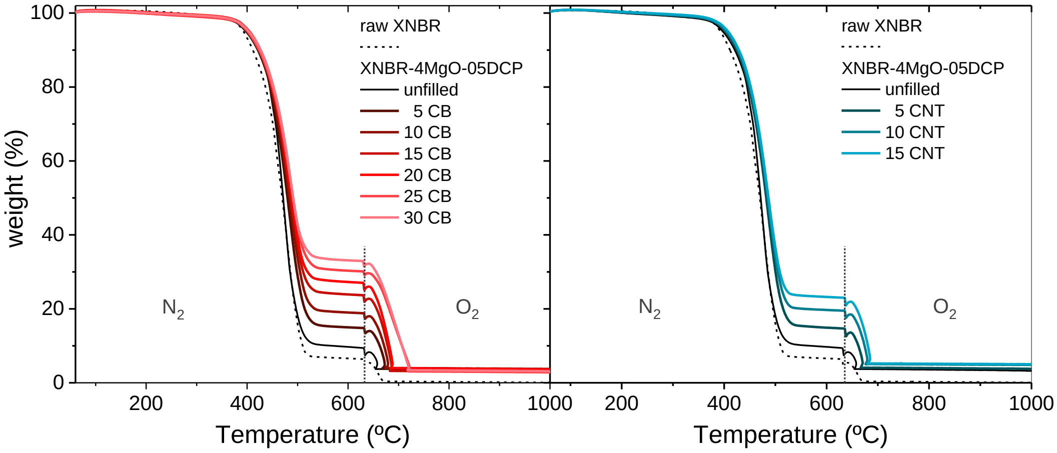

Figure S1. Thermograms of elastomeric samples between 50 and 1000 ° C at 30 ° C / min in which the degradation of the matrix and the decomposition of the carbon black and carbon nanotubes fillers is observed in an N2 (up to 650 ° C) and O2 atmosphere (up to 1000 ° C).

Comparing the results obtained from the TGA of the vulcanized samples with the filler content introduced in the preparation of the different compounds (raw, before their vulcanization), we obtain the data reflected in Figure S2.

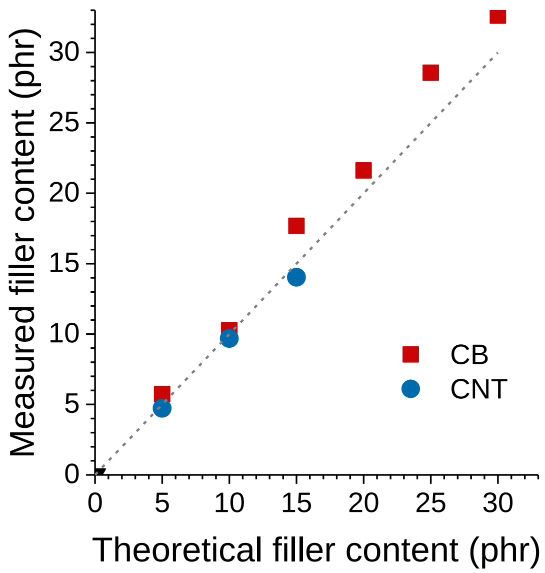

Figure S2. Relationship between the theoretical content introduced in the formulation of the samples and the actual filler content calculated by TGA. Dashed line shows a 1:1 ratio.

Therefore, the incorporation of the filler to the elastomeric compound is totally effective and it is possible to characterize the variation of its properties as a function of these once the pristine sample has been previously characterized.

FEG-SEM

To visualize the size and distribution of fillers in the elastomeric XNBR matrix samples were analyzed by FEG-SEM (Nova NanoSEM FEI 230). Representative images for carbon black and multiwalled carbon nanotubes filled samples are shown in Figures S3 and S4 respectively.

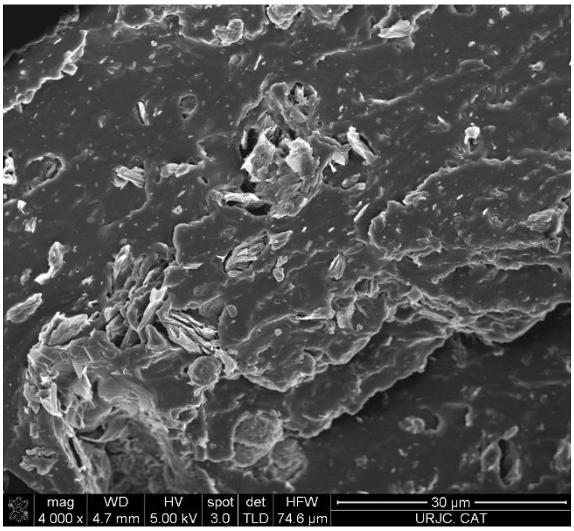

Figure S3. FEG-SEM image of sample "10CB" at low magnification (4000x)

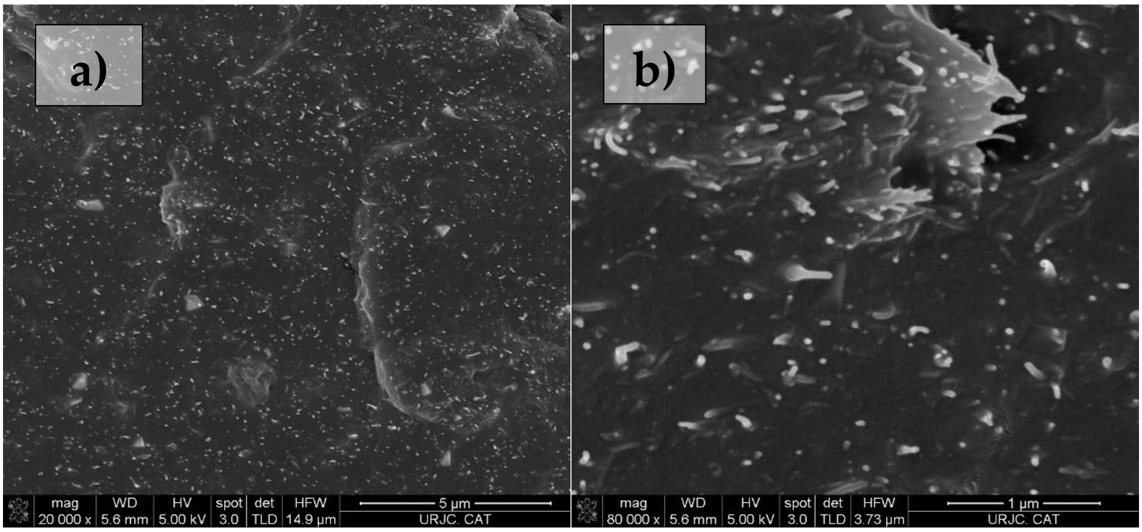

Figure S4. FEG-SEM images of sample "10CNT" at high magnification [a) 20000x, and b) 80000x.]

DMA data results

Table S1. Data results obtained from DMTA study of the samples. T_g bulk and T_{ionic} were obtained as the maximum of the tan δ peaks in the study.

Sample	*E'* at – 25 °C	*E' at* 25 °C	T_g bulk	T_{ionic}
	MPa	MPa	ºC	ºC
**Unfilled**	2900	12.0	-5.3	66.9
**5CB**	2113	15.2	-4.6	66.8
**10CB**	2442	22.9	-4.1	74.1
**15CB**	2597	32.4	-3.7	73.8
**20CB**	2811	54.5	-3.9	79.8
**25CB**	2890	75.7	-4.6	86.7
**30CB**	3009	122.5	-4.8	82.0
**5CNT**	2319	22.5	-3.2	80.1
**10CNT**	2780	42.6	-2.9	82.8
**15CNT**	3385	92.5	-2.7	86.0

Shape-memory data results

Table S2. Fixing (R_f) and recovery (R_r) ratios of the shape memory for 4 consecutive cycles in each sample.

Sample		R_f				R_r		
		%				%		
Cycle:	1	2	3	4	1	2	3	4
**Unfilled**	78.6	80.4	81.4	82.1	81.5	86.6	92.6	95.2
**5CB**	81.1	81.5	82.0	82.2	91.5	96.0	96.0	96.8
**10CB**	85.1	85.8	86.2	86.5	87.8	92.7	94.9	95.3
**15CB**	85.0	85.4	85.7	86.0	84.7	94.5	95.5	95.9
**20CB**	86.6	87.2	87.4	87.7	91.8	93.9	94.8	95.3
**25CB**	86.7	87.8	87.9	88.0	85.3	89.5	93.3	94.8
**30CB**	87.9	88.4	88.7	89.0	75.2	85.3	91.1	93.3
**5CNT**	82.4	83.1	83.5	83.9	90.2	95.6	96.8	97.0
**10CNT**	86.6	87.4	87.9	90.0	90.1	93.1	94.6	94.6
**15CNT**	89.3	90.1	90.3	90.6	86.9	92.0	95.1	95.5
